# New SPM8-based MRAC method for simultaneous PET/MR brain images: comparison with state-of-the-art non-rigid registration methods

**DOI:** 10.1186/2197-7364-1-S1-A29

**Published:** 2014-07-29

**Authors:** David Izquierdo-Garcia, Kevin T Chen, Adam E Hansen, Stefan Förster, Didier Benoit, Sylvia Schachoff, Sebastian Fürst, Daniel B Chonde, Ciprian Catana

**Affiliations:** Athinoula A. Martinos Center for Biomedical Imaging, Department of Radiology, Massachusetts General Hospital and Harvard Medical School, Charlestown, MA USA; Department of Health Sciences and Technology, Massachusetts Institute of Technology, Cambridge, MA USA; Department of Clinical Physiology, Nuclear Medicine and PET, Rigshospitalet, Copenhagen University Hospital, Copenhagen, Denmark; Department of Nuclear Medicine, Technische Universität München, Munich, Germany; Program in Biophysics, Harvard University, Cambridge, MA USA

We describe a new MR-based attenuation correction (MRAC) method for neurological studies performed using integrated PET/MR scanners. The method, combining the advantages of image segmentation and atlas-based approaches to generate a high-resolution template, is based on the widely available SPM8 software and provides robust and accurate linear attenuation coefficients (LACs) for head while requiring minimal user interaction.

*Atlas generation*: 3T MR and CT images from 15 glioblastoma subjects were used to generate the high-resolution atlas. MR images were segmented into 6 tissue classes: GM, WM, CSF, soft tissue, bone and air)[1]. Tissue classes were then coregistered using an iterative diffeomorphic image registration algorithm [2] to form the template.

*Atlas validation*: The template was validated on 16 subjects. SyN [3] and IRTK [4], considered state-of-the-art for non-rigid image registration[5], were used for comparison. Final attenuation maps were created from the warped CT atlas following [6]. PET images were then reconstructed using the proposed methods as well as the manufacturer’s built-in method (dual-echo Dixon-VIBE sequence) [7] and compared to the gold standard CT-based attenuation correction (CTAC).

**Figure 1:**
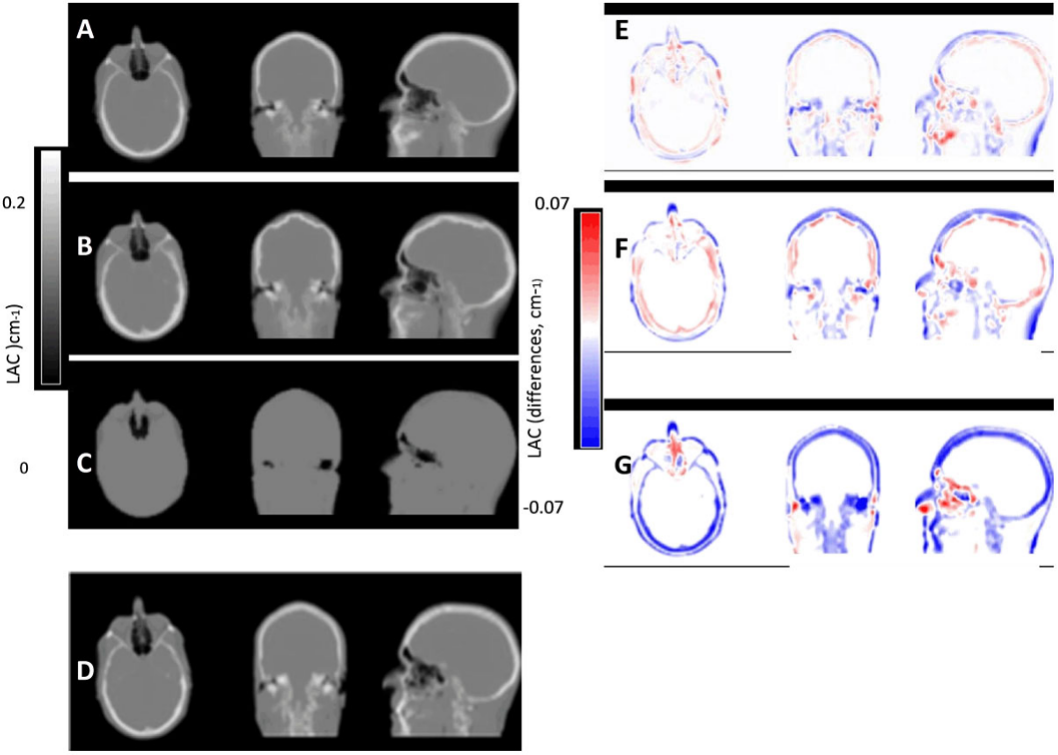
Comparison of LACs from a validation subject for our proposed method (A), the SyN method (B) and the manufacturer’s built-in Dixon method (C) to the gold standard CTAC (D). Image differences with respect to the gold standard CTAC of our method (E), the SyN method (F) and the Dixon method (G).

**Figure 2:**
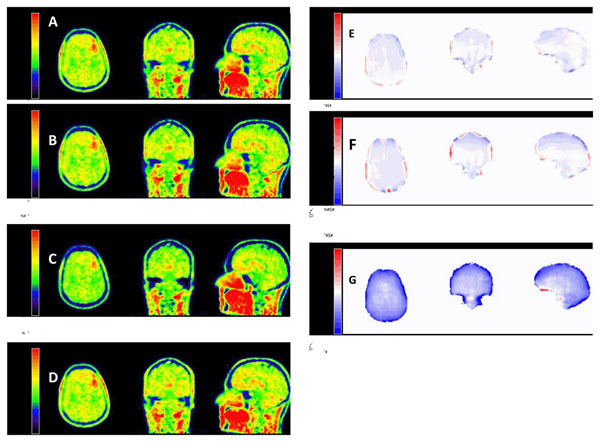
PET images from a validation subject reconstructed with our proposed method (A), with the SyN method (B) and with the manufacturer’s built-in Dixon method (C), compared with the gold standard CTAC (D). Relative changes (in % with respect to gold standard, CTAC) for our method (E), the SyN method (F) and the Dixon method (G).

The qualitative and quantitative analysis of the attenuation maps revealed that the SPM8-based method produces very robust results (Figure 1). In terms of the PET data quantification, we observed improvements of > 70% compared to the VIBE-based method (Table 1 and Figure 2). When compared to SyN-based image registration, the SPM8 approach showed improved global results on the brain area (Figures 1 and 2).

**Table 1:** Summary of voxel- and ROI-based results between our method (atlas) and the current manufacturer’s method (Dixon)

		*	*	*	*
		*µatlas*	*µDixon*	*PETatlas*	*PETDixon*
Validation	*All ROIs*	**0.99(1.81)**	**3.04(3.15)**	**2.74(2.28)**	**9.38(4.97)**
dataset	*Voxel-based*	**1.86(4.06)**	**4.18(6.68)**	**3.87(5.0)**	**13.0(10.25)**

^*^ Average across subjects of the mean absolute relative changes, RC(%) and SD: Mean(SD);

We presented a new MRAC technique for brain images acquired on simultaneous PET/MR scanners. The new approach relies on segmentation- and atlas-based features to provide robust and more accurate LACs than using state-of-art non-rigid image registration while avoiding sophisticated user input or interaction.
